# Neuropsychological function in individuals with morbid obesity: a cross-sectional study

**DOI:** 10.1186/s40608-017-0143-7

**Published:** 2017-01-26

**Authors:** Hanna L. Sargénius, Stian Lydersen, Knut Hestad

**Affiliations:** 1 Department of Psychology, Faculty of Social Sciences and Technology Management, Norwegian University of Science and Technology (NTNU), Trondheim, Norway; 2 Centre for Old Age Psychiatric Research, Innlandet Hospital Trust, Postboks 68, Ottestad, 2313 Norway; 3 Regional Centre for Child and Youth Mental Health and Child Welfare, NTNU, Trondheim, Norway; 4 Department of Research, Innlandet Hospital Trust, Brumunddal, Norway; 5 Department of Public Health, Hedmark University of Applied Sciences, Elverum, Norway

**Keywords:** Morbid obesity, Executive function, Neuropsychological profile, Deficit score

## Abstract

**Background:**

Previous research has shown cognitive dysfunction to be present in a significant number of individuals with obesity. The objective of this study was to assess the neuropsychological profile of morbidly obese patients referred to weight-loss treatment.

**Methods:**

An extensive battery of neuropsychological tests with well-known normative data covering various cognitive domains was administered to 96 patients. The test results were transformed to z-scores for comparisons with normative data. As a means of determining level of cognitive impairment *within* the group, deficit scores were applied. Group comparisons on the different cognitive domains were conducted between patients with depressive symptoms and patients reporting no such symptoms.

**Results:**

As illustrated in mean z-scores, the patients demonstrated lower performance compared to normative data on visual memory (mean -.26, CI -.43 to -.09, *p* = .003), speed of information processing (mean -.22, CI -.34 to -.09, *p* = .001), executive functions (mean -.28, CI -.40 to -.16, *p* < .001), and attention/vigilance (mean -.25, CI -.37 to -.13, *p* < .001). Their performance was good on verbal fluency (mean .24, CI .04 to .44, *p* = .016) and verbal memory (mean .55, CI .38 to .72, *p* < .001). No significant performance differences were observed in the cognitive domains of visuospatial ability, motor function, and working memory. The deficit scores, however, revealed working memory and motor function to be significantly impaired within the group as well. Patients with depressive symptoms differed from patients without such symptoms on visual memory (mean .43, CI .07 to .80, *p* = .021).

**Conclusions:**

Some characteristic cognitive weaknesses and strengths were evident at the group level, although pronounced variation was observed. Deficits in executive functions, information processing, and attention should be taken into consideration in clinical practice.

## Background

Obesity is a complex condition, encompassing both physiological and emotional entities [1]. Why some individuals are predisposed for severe obesity and others don’t is not fully understood. Genetic and environmental factors have been provided as the major causes for our growing waistline. Recent attention has shifted towards the view that differences in the brain could be both a consequence of, and an explanatory factor for obesity. A growing body of structural brain imaging studies taking advantage of CT, SPECT, and fMRI has demonstrated that, compared with their normal-weight counterparts, middle-aged and otherwise healthy elderly adults with obesity are likely to demonstrate decreased blood flow in the prefrontal cortex [2]; express greater cerebral glycolytic metabolism, especially in the posterior cingulate gyrus [3]; and demonstrate increased temporal lobe atrophy and white matter disease [4–6]. More precisely, both overweight (body-mass index [BMI] = 25–30) and obese (BMI > 30) individuals seem to be affected by atrophy in a wide range of brain regions, although this effect is more prominent in the obese group [7]. There is currently a common understanding that both obesity and the duration of obesity have adverse consequences for the brain, providing us with a possible explanation for the greater risk of late-onset Alzheimer’s disease (AD) found in obese individuals [7].

Elevated body mass and central (abdominal) obesity have been repeatedly linked to cognitive impairment across the life span [8, 9]. Attention, memory, and executive function seem to be the most affected cognitive domains, a situation that can also occur independent of medical comorbidities in adults and adolescents [9–12]. Interestingly, intentional weight loss has been seen to reverse cognitive deficits to a certain extent, especially on the domains of memory [13, 14] and executive function [15]. This trend has been observed only in obese individuals, however, and has not been documented in cases involving subjects who are merely overweight [16]. The relationship between obesity and cognition is even more complex, as the composition of adipose tissue changes over the lifespan [17]. Higher levels of body weight in old age is associated with a *lower* risk of AD and apparently slows down the clinical progression of cognitive decline [18–20]. Our knowledge of cognitive dysfunction in obese individuals is inconclusive and limited, as the complexity of obesity as a condition introduces many methodological concerns [21]. Neuropsychological functions in relation to obesity have most often been examined with relatively few tests of cognitive abilities at one administration.

The aims of our study are to explore cognitive function in mid-life obesity and the effect of depressive symptoms on cognition in greater detail. By using a wider array of tests, the goal is to construct a neuropsychological profile for this clinical group. Although similar efforts have been made within the eating disorder literature [22, 23], data of this type remains limited relative to obesity. Furthermore, we wanted to determine levels of neuropsychological impairment among our patients by means of deficit scores, which, in contrast to using only standardized test scores, will give us the ability to differentiate subjects on the basis of mild, moderate, and severe neuropsychological deficits [24].

## Methods

### Study sample and geographical area

Patients diagnosed with morbid obesity and referred to one of two obesity units within Innlandet Hospital Trust were invited to participate in this study consecutively, from September 2013 to March 2015. Inclusion criteria were a) eligibility for morbid obesity (MO) treatment according to public health-care guidelines, b) having a BMI ≥ 40 kg/m^2^ or a BMI ≥ 35 kg/m^2^ with the presence of a comorbidity, c) having completed ≥ 8 years of education, and d) being between 18 and 60 years of age. A total of 97 patients aged 18 to 60 gave their written informed consent to participate; one patient that did complete the inclusion and assessment was later excluded for medical reasons, leaving 96 patients (74 female) in the analysis. Exclusion criteria were a) history of neurological disorder or injury (e.g. dementia, stroke, or seizures), b) moderate or severe head injury (defined as >10-min loss of consciousness), c) past or current history of severe psychiatric illness (e.g. schizophrenia, bipolar disorder), d) past or current history of alcohol or drug abuse, e) history of a learning disorder, or f) developmental disabilities.

Two obesity units— the only two in this part of Norway — provide health care services mainly to patients residing in the Hedmark and Oppland counties in the south-east part of the country with approximately 400 000 inhabitants. Because the Free Hospital Choice Norway Program allows patients to choose the obesity unit they prefer, however, these units also serve visiting patients from other parts of Norway. Comparisons between our sample and randomly selected patients from Hedmark or Oppland who were receiving health-care services for their obesity at these units could reveal no significant differences in age, weight, or anthropometric measures (data not shown). Thus our sample for this study, with mean (SD) age of 43.55 (9.25) years and a mean BMI of 42.62 (5.52) kg/m^2^ could be considered representative of this clinic population. See Table 1 for more detailed descriptions.

**Table 1:** Patient descriptive

	N	Mean	SD	Minimum	Maximum
Age	96	43.55	9.25	18	60
BMI	88	42.61	5.52	31.8	59.7
WHR	87	0.96	0.10	0.46	1.17
SBP	83	139	15.58	115	187
DBP	83	85.9	8.46	60	122
Verbal comprehension index^a^	94	90.73	11.18	68	122
Perceptual organization index^a^	96	106.55	14.43	69	145
BDI-II raw score	95	10.0	9.22	0	38
BDI-II cognitive subscale	94	3.91	4.54	0	19
BDI-II non-cognitive subscale	94	6.14	5.44	0	22

*Notes: BMI* body mass index, (weight/m^2^), *WHR* waist-to-hip ratio, (waist/hip), *SBP* systolic blood pressure, *DBP* diastolic blood pressure, *BDI-II* Beck Depression Inventory II

^a^Figures are derived from the Wechsler Adult Intelligence Scale III

### Material and neuropsychological assessment

The test battery we selected was based upon earlier research conducted in populations of individuals with both morbid obesity and eating disorders, and comprised 15 tests with a total of 43 outcome variables. The Delis-Kaplan Executive Function System [D-KEFS] provided the Tower Test (total achievement score, precision score), Color Word Interference Test [CWIT] (color naming, word reading, inhibition, inhibition/switching), and Verbal Fluency Test [FAS] (letter fluency, category fluency, category switching). The Wechsler Memory Scale Revised [WMS-R] supplied the Digit Span Test (forward and backwards), Logical Memory Test (Immediate and delayed recall), and Visual Memory (Immediate and delayed recall). The Halstead-Reitan Battery provided the Trail Making Test (Part A and B), Dynamometer, Grooved Pegboard Test, and the computerized Category Test 108-card version (total correct). We also administered the Wisconsin Card Sorting Test [WCST] 64 Computer version (total errors, perseverative responses), Conner’s Continuous Performance Test II [CPT-II], Paced Auditory Serial Addition Test [PASAT] (3 and 2 s interval), California Verbal List Learning Test II [CVLT-II] (total recall across trials, short free recall, delayed free recall), and Rey-Complex Figure Test [RCFT] (copy, immediate recall, delayed recall). To find estimates of intellectual function, subtests from the Wechsler Adult Intelligence Scale 3rd Edition [WAIS-III] were used to find Verbal Comprehension Index (Vocabulary, Similarities, and Information), Perceptual Organization Index (Picture Completion, Block Design, and Matrix Reasoning), and Processing speed (The Digit Symbol). The Norwegian-translated versions of these tests have been shown to retain their central psychometric properties, with several comparative studies confirming that the majority of tests reveal only minor differences between Norwegian and US test-performance scores [25–28].

### Clinical assessment

Patients were screened for depression (Beck Depression Inventory II), fatigue, (Fatigue Severity Scale), sleepiness (Epworth Sleepiness Scale), psychological stress (Hopkins Symptoms Check List 10), self-esteem (Rosenberg Self-esteem Scale), humor (Sense of Humor Questionnaire-6), and psychological well-being (World Health Organization Five Well-being Index). Information regarding current medical conditions was obtained from self-reports and from patients’ medical records. This comprehensive assessment was undertaken in order to provide additional information that could be useful when interpreting our data and to function as a reference on follow-up of patients. The total procedure, including clinical and neuropsychological testing, lasted in approximately 4.5 h.

### Ethics

Approval was obtained from the Regional Committee for Medical and Health Research Ethics (REC) Norway in July 2012 – Reference 2012/966. All patients sought treatment voluntarily, and were notified that their test results would have no implications for their further treatment provision. All participants gave their written informed consent before being included in the study.

### Data management

Raw scores on the neuropsychological tests were standardized by using the appropriate demographic normative data for the respective test manuals, correcting for age, sex, and education. For the RCFT Copy trial measure only, we additionally included a group of 98 healthy US controls (MMSE: mean 28.81 (SD) 1.190) similar to our sample in age (mean 41.45 (SD) 11.77) and education for comparison with the clinical sample.

For the purpose of data analysis, the direction of scores was reversed on tests on which higher scores represent poorer performance (e.g. CPT-II). All scores were converted into z-scores (mean = 0, SD = 1) for the main analysis. Composite indices for the cognitive domains visual memory, verbal memory, processing speed, visuospatial ability, working memory, verbal fluency, executive function, and attention/vigilance were created by summarizing and averaging the z-scores from each of the specific tests within that domain, except for the visuospatial domain, in which the RCFT Copy measure was not included, but was handled as an independent score (see Table 2).

**Table 2:** Demographically adjusted performance z-scores with cognitive domains

Measure	Mean (SD)	Minimum	Maximum	*t*-value
CVLT Total recall	.695 (1.406)	−4.720	3.8	
CVLT Immediate free recall	.394 (1.117)	−3.500	2.000	
CVLT Delayed free recall	.415 (1.020)	−2.500	2.000	
WMS-R Verbal memory 1	.567 (.871)	−1.881	2.326	
WMS-R Verbal memory 2	.688 (.883)	−2.326	2.326	
Verbal memory mean	.552 (0.842)	−2.011	2.256	6.391**
WMS-R Visual memory 1	.291 (.805)	−1.555	2.054	
WMS-R Visual memory 2	-.226 (.875)	−2.326	2.326	
RCFT Immediate recall	-.532 (1.199)	−4.900	1.600	
RCFT Delayed recall	-.568 (1.321)	−4.900	1.800	
Visual memory mean	-.259 (.838)	−2.692	1.895	−3.024*
D-KEFS CWIT Color naming	-.382 (.807)	−3.333	1.000	
D-KEFS CWIT Word reading	-.115 (.707)	−2.333	1.000	
WAIS-III Digit symbol	-.326 (.742)	−1.667	2.000	
TMT A	-.046 (1.256)	−5.000	2.100	
Processing speed mean	-.219 (.595)	−1.825	.908	−3.567*
WAIS III Block design	.340 (.957)	−1.667	3.000	
WAIS III Matrix reasoning	.361 (.875)	−1.667	2.333	
RCFT Copy^a^	-.036 (1.067)	−3.505	1.549	
Visuospatial ability mean	.152 (.810)	−1.766	1.835	1.838
WMS-R Digit span forward	.308 (.889)	−2.326	2.326	
WMS-R Digit span backward	.882 (1.183)	−1.645	2.326	
PASAT3	-.190 (1.015)	−3.274	1.529	
PASAT2	−1.057 (.988)	−4.107	.794	
Working memory mean	.002 (.658)	−1.418	1.450	.033
WCST Total error	-.670 (.993)	−3.000	2.000	
WCST Perseverative responses	-.503 (.777)	−2.700	2.900	
D-KEFS Tower total correct	.203 (.701)	−2.000	1.667	
D-KEFS Tower precision score	-.284 (.901)	−2.667	3.000	
TMT B	-.167 (1.246)	−3.900	1.800	
D-KEFS CWIT Inhibition	.063 (.848)	−3.000	1.333	
D-KEFS CWIT Inhibition/switching	-.182 (1.015)	−3.000	1.333	
Executive function mean 1 (Tower total)	-.198 (.572)	−2.272	1.267	−3.290*
Executive function mean II (Tower precision)	-.283 (.551)	−1.772	1.156	−4.867**
D-KEFS Letter fluency	.020 (1.230)	−2.333	3.000	
D-KEFS Category fluency	.677 (1.347)	3.000	6.000	
D-KEFS Category switching	.031 (946)	−2.333	2.333	
Verbal fluency mean	.243 (.967)	−2.333	2.444	2.461**
(CPT-II) Omissions	-.455* (1.176)	−4.620	.790	
Commissions	-.552 (1.172)	−3.689	1.429	
Hit rate	.470 (1.275)	−3.876	3.398	
Hit RT SE	-.565 (1.346)	−4.985	2.010	
Detectability	-.429 (.865)	−3.302	1.674	
Hit RT block change	-.266 (1.087)	−3.172	2.584	
Hit SE block change	-.898 (1.311)	−7.590	1.456	
Hit RT ISI change	-.034 (1.301)	−4.247	2.404	
Hit SE ISI change	.009 (1.356)	−4.462	2.672	
Attention/vigilance mean	-.251 (.557)	−1.50	.76	−4.254**
Dynamometer Dominant hand	-.109 (1.058)	−2.400	2.300	
Dynamometer Non-dominant hand	.023 (1.076)	−1.700	3.200	
Pegboard Dominant hand	-.247 (1.147)	−5.000	2.100	
Pegboard Non-dominant hand	-.333 (1.329)	−5.000	1.700	
Motor function mean	-.154 (.780)	−1.750	1.350	−1.802

Notes: No normative data was available for the Category test 108-card version. Thus this test was left out from further analysis

^a^RCFT Copy: A US sample of 98 healthy controls similar in age and years of education were used as reference

*p-value < .05

**p-value < .001

Deficit scores for each test score were also created as a means of determining level of cognitive impairment within the group. The deficit scores are constructed from the same performance raw scores used previously in the main analyses. Rather than using z-scores, however, we used demographically corrected T-scores to provide impairment ratings on a 5-point scale. A deficit score of 0 is considered normal performance (T-score ≥ 40), a deficit score of 1 equal to mild impairment (T-score = 35–39), 2 to mild-to-moderate impairment (T-score = 30–34), 3 to moderate impairment (T-score = 25–29), 4 to moderate-to-severe impairment (T- score = 20–24), and 5 to severe impairment (T-score <20). Domain Deficit Scores (DDS) were then constructed by averaging the deficit scores within the various cognitive domains. Participants were classified as impaired if the average of their impairment scores were greater than .5 on a particular DDS. A Global Impairment Score was then derived by averaging the DDS across all tests in the battery. As with the DDS impairment ratings, patients were classified as having Global Impairment if they had an average deficit score above .5. The use of the “deficit score” approach reflects our focus on abilities that may have been affected by CNS injury or disease. It has been used in many previous studies, for instant summarizing neuropsychological data has been used to detect relatively “spotty” and sometimes subtle impairment associated with HIV infection [29–31], schizophrenia [32], and has shown good sensitivity and specificity in predicting gold standard classifications by expert clinical ratings [29, 31]. The GDS approach using a single cut-off for detecting impairment appears to generalize to different patient groups and even largely different test batteries [31]. Similar to clinical ratings of neurocognitive results, which do not let good/normal performances on some tests or domains obscure problematic results on others, purposely “ignores” test scores in the normal range [24, 33].

### Statistical analysis

A series of one-sample *t*-tests were computed in order to determine whether the scores for the patient group differed significantly from the normative test mean, and independent-sample *t*-tests were performed between the patient group and the healthy controls on the RCFT Copy, and between the depressive symptom patient group and the non-depressive symptom patient group. Normality of residuals was checked by visual inspection of Q-Q plots. Some of the variables displayed moderate deviations from normality. In these cases, we also computed *p*-values and confidence intervals (CI) using Bootstrapping with the bias-corrected and accelerated method (BCa) for 5000 bootstrap samples. These analyses gave essentially the same results as the *t*-test-based *p*-values and CIs.

Participants were excluded from a specific analysis if their data were not available for a relevant test. Because the rules of administration for some of the neuropsychological tests (e.g. CWIT) required that the test to be stopped if the patient did not achieve a high enough score to reach the next level, cases had to be excluded analysis by analysis rather than listwise.

Pearson’s correlation and multiple linear regression was used to examine the possible relationship between obesity, depressive symptoms, and cognitive function. Statistical significance was set at *p* < .05, and 95 percent CI are reported where relevant. SPSS 24.0 (IBM Corporation, Armonk, NY, USA) was used in all statistical analysis.

## Results

**Fig. 1:**
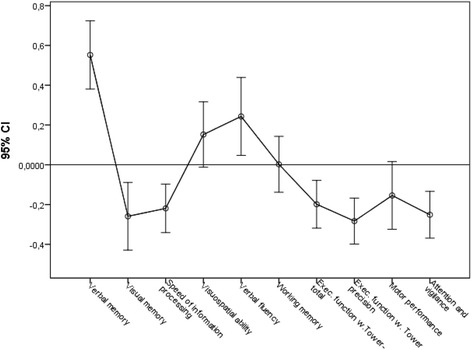
The neuropsychological profile of the MO patients. Illustrated by mean z-scores on the cognitive domain indices and 95% CIs

**Fig. 2:**
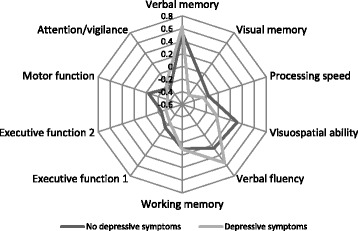
Radar chart illustrating the performance of the depressive symptoms and non-depressive symptoms groups for the different cognitive domains

Demographically adjusted performance scores for each individual test at the group level are presented in Table 2. The mean z-scores on the cognitive domain indices and 95% CIs are illustrated in Fig. 1. After constructing the composite indices for each domain, the patients’ performance remained significantly worse than the normative means z-scores on visual memory (mean -.26, CI -.43 to -.09, *p* = .003), speed of information processing (mean -.22, CI -.34 to -.09, *p* = .001), executive function (Executive function with Tower total achievement: Mean -.20, CI -.31 to .07, *p* = .001; Executive function with Tower precision: Mean -.28, CI -.40 to -.16, *p* < .001), and attention/vigilance (mean -.25, CI -.37 to -.13, *p* < .001). In contrast, the patients performed better than the norms on verbal fluency (mean .24, CI .04 to .44, *p* = .016) and verbal memory (mean .55, CI .38 to .72, *p* < .001). Furthermore, their performance was well within normal range on motor function (mean -.15, CI -.32 to .01, *p* = .075), visuospatial ability (mean .15, CI -.01 to .31, *p* = .069), and working memory (mean .002, CI -.13 to .14, *p* = .974). On the RCFT Copy trial, an independent sample *t*-test revealed no difference between the MO-patients (mean 30.19, SD = 3.85) and the healthy controls (mean = 30.33, SD = 3.66) on the figure copying; *t* (189) = 2.55, *p* = .811. Group comparisons between younger and older adults, with a cut-off value set to 40 years of age did not reveal any significant difference in cognitive performance. Pearson’s correlations revealed no significant relationships between test performance and measures of obesity in terms of BMI or waist-to-hip ratio (WHR). Depressive symptoms correlated significantly with visual memory (*r* = −.26, *p* = .012), and information processing (*r* = −.21, *p* = .038). Patients with depressive symptoms differed from patients without such symptoms on visual memory (mean .43, CI .07 to .80, *p* = .021). For an overview of the cognitive profiles of the depressive symptoms group versus the noon-depressive symptoms group see Fig. 2.

**Fig. 3:**
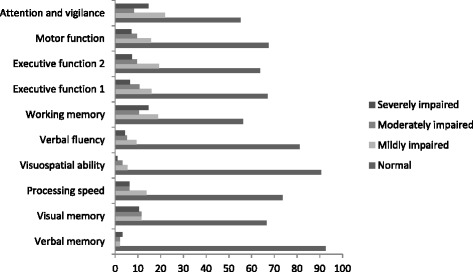
Distribution of DDS levels. Patients sorted according to being within normal range, mildly impaired, moderately impaired, and severely impaired. Numbers reported in %

The percentage of patients classified as cognitively impaired (GDS ≥ .50) was 18.7%. For the assorted cognitive domains, the percentages of impaired patients (DDS ≥ .50) were 7.4% on verbal memory, 33.3% on visual memory, 26.3% on processing speed, 9.4% on visuospatial ability, 18.7% on verbal fluency, 33.3% on working memory, 33.0% and 36.2% on Executive function (with Tower total correct/with Tower precision score), 32.5% on motor function, and 44.8% on sustained attention/vigilance. Figure 3 presents the distribution of DDS impairment levels.

## Discussion

We examined the cognitive performance of a sample of patients with morbid obesity in an effort to construct a neuropsychological profile. We observed neuropsychological inefficiencies in our sample related to executive function, attention/vigilance, processing speed, and visual memory, albeit with great within-group variation. Additional inefficiencies in working memory and motor function were revealed when we applied the deficit scores to each individual’s test performance. Most participants performed within the normal range, but there were subgroups that displayed specific cognitive difficulties.

Significantly, patients performed within normal range on visuo-constructional tasks, although with difficulty on immediate and delayed recall memory. Although this finding supports reduced visual memory in obesity [10], it contradicts other studies finding a relationship between obesity and visuo-constructional performance [10, 12]. It is noteworthy that our sample’s poor performance on the delayed recall tasks cannot be explained by poor memory per se, as verbal memory was exceptionally good. Perhaps this reduced performance on the visual recall conditions is related to the extent to which the visual material in question was organized and processed. Among anorexia nervosa (AN) and bulimia nervosa (BN) patients, a focus on details rather than on gestalt features arguably explains good accuracy scores followed by poor performance on visual tasks. A similar detailed-focus style could be present among obese people as well. To the best of our knowledge, only one study has investigated this idea and has found support for it [34]. We propose that a distorted self-perception of one’s body size, shape, and placement in space is enhanced by two factors: an inefficient organizational strategy for spatial material and an inability to see the complete gestalt. This “size blindness” may lead to further accumulation of unwanted excess weight, a possibility requiring further investigation.

We also constructed two separate composite scores representing the domain of executive function, based upon a recommendation to use the Tower test precision score rather than the total correct score when applying this neuropsychological test [22]. Both composite scores, therefore, comprise a measure from the Tower test: a precision score and a total correct score. Although both composite scores revealed dysfunction in this cognitive domain, the precision score appeared to be a better measure of actual functioning level, as substantiated by our findings regarding increased impulsivity and reduced attention spans. The actions of our patients were impulsive (e.g. on the CPT-II), with lack of attention to neatness across tests (e.g. on the WMS-R visual memory tasks). They often demonstrated low motivation to complete the task, particularly on the Tower test or the PASAT (the test most subjects declined to finish). Problems with impulsivity are suggested to be a common trait among overweight and obese individuals. A deficit in any aspect of executive function, such as intrusive thoughts and the inability to inhibit automatic or dominant behaviors [35] may contribute to challenges with the development of alternative coping strategies, rendering these patients more vulnerable to employing unhealthy eating behaviors in response to emotional distress [12].

Our findings are largely in line with research demonstrating comparable results with individuals with both AN and BN, especially on executive functioning [22, 23, 36]. Whether or not the endocrine effect of adipose tissue on the central nervous system plays the major part in the relationship between cognition and body weight [37], Fagundo et al. suggest that cognitive functioning in extreme weight conditions like eating disorders and morbid obesity have similar dysfunctional executive profiles [36], a suggestion documented in meta-analyses [38, 39]. Although findings are inconsistent, it has been suggested that starvation or caloric restriction affect cognitive performance, particularly psychomotor speed and executive function [40]. Perhaps malnutrition in the context of obesity is operating in pathways similar to those seen in eating disorders; proper nutrition, it has been suggested, could protect people from accelerated cognitive decline [19, 41].

Given this hypothesis, we examined whether degree of obesity, measured by body mass index and waist-to-hip ratio, had any associations with cognitive task performance in our study, and found no such relationships. Perhaps the BMIs of our patients were not high enough relative to the sample size, and we had insufficient statistical power to capture a significant difference. Although the mean BMI in our sample classifies our subjects in morbid obesity class III, BMI ranged from obesity class I to III. Furthermore, nadir BMI has been found in AN and BN patients to be a more powerful mediator for cognitive test performance than the BMI is [23]. Perhaps similar to patients with eating disorders in which an extremely low BMI may lead to a longer-lasting reduction in cognitive functioning, the highest BMI acquired by a patient may have similar effects in obesity. Alternatively, patients with premorbid cognitive difficulties may develop more severe obesity, rather than vice-versa. Nonetheless, the neuropsychological performance of our group revealed weaknesses on several cognitive tests when compared to normative data, particularly when the deficit scores were analyzed. A deficit score approach is recognized as an efficient and reliable way to locate neurocognitive and functional deficits within individuals when the goal is to identify more severe levels of cognitive impairment [24]. Our findings indicate that our patients expressed specific cognitive inefficiencies rather than global impairment. Such cases will not likely be detected in standard enrolment in weight-loss treatment programs, which, in turn, may obstruct successful treatment outcomes. A patient can appear to be within normal functioning level, but a different story may emerge under scrutiny. The majority of our patients were within normal functioning level when using the GDS, but when studying each DDS, almost half of could be classified as mildly impaired (DDS = .50–.90) – or worse in some of the cognitive domains. More alarming, in all cognitive domains we found patients who were both moderately (DDS = 1.0–1.39) and severely impaired (DDS = > 1.40). Visual memory, working memory, and attention/vigilance are of greatest concern among our patients according to DDS. We believe this method to have good clinical value. The balance between sensitivity and specificity in classifying cognitive dysfunction at a cut point of ≥ .50 has repeatedly been supported by previous studies using different subject samples and cognitive test batteries, all confirming a good generalizability of this method (e.g. Blackstone et al.). For instance, Heaton et al. [33] performed GDS for normal healthy subjects using an expanded Halstead-Reitan Battery. They found 11.3% of their subjects to score within the mildly impaired range, 0.91% within the mildly to moderately impaired range, and 0.13% scored in the moderately impaired range. Thus, health providers must be encouraged to increase their awareness about the most afflicted domains and be mindful of the way cognitive inefficiencies can affect treatment outcomes. A reduction in cognitive performance could be expected if there are increasing risk factors related to age. However, cognitive deficits seem to be present in obesity independently of age. Our analyses could not reveal any differences between younger and older adults within our sample, despite a wide age range. Furthermore, it is noteworthy that in our study, only visual memory performance separated the depressed patients from the non-depressed. Apart from this cognitive domain, the neuropsychological profiles for the two groups were the same. This supports the presence of cognitive dysfunction in this group, regardless of depressive symptoms. It is easy to focus on the patients who struggle with mood disturbances, but just as much, patients with apparently no depressive symptoms need to be supported. Nevertheless, depression is of some importance regarding cognitive functioning in this patient group.

A key strength of this study is its large sample of both males and females. Furthermore, we employed an extensive neuropsychological test battery comprised entirely of well-established tests covering a wide range of cognitive domains. Although we did not use the full-scale IQ, we did include two indices of intellectual functioning in our analyses.

One limitation of this study that weakens the results is the use of normative comparison data rather than a control group for the standardized measures. Nonetheless, published norms are considered representative of sociodemographic characteristics at the population level. Second, we did not adjust for the presence of the comorbidities that most often follow obesity, our rationale being that patients presenting for clinical treatment at specialist centers most likely come from selected subgroups significantly differing from other populations in the first place [22]. Indeed, many patients receive treatment *because* of their comorbidity. Moreover, the various comorbidities are extremely diverse, yet strongly intertwined and difficult to separate. Third, we could be criticized for not considering the issue of multiple comparisons. We believe that our work provides a valuable piece to the jigsaw puzzle of obesity, however, as we take comfort in Senn’s statement that the methodological controversy still remains among scientists within the field of multiplicity adjustment [42] and that adjusting for multiplicity has been argued against in many settings [43, 44].

## Conclusion

The research hypothesis concerning the relationships between obesity and cognitive function is not new, however, it is a need to specify if this clinical group’s cognitive deficits represents a general cognitive decline or if it is more specific. This study supports preceding work finding subjects with morbid obesity to exhibit some cognitive weaknesses on executive function, memory, processing speed, and attention/vigilance. The tests we used clearly show that the cognitive deficits are more like specific learning disorders. These cognitive challenges may be distinctive for individuals with severe obesity, and may therefore be the actual reason why many patients fail during treatment. We argue that treatment could be better individualized and that a successful treatment outcome could be better facilitated by obtaining a neuropsychological profile with complementary deficit scores of patients seeking weight-loss treatment.

## Abbreviations

AN: Anorexia nervosa
BMI: Body mass index
BN: Bulimia nervosa
DDS: Domain deficit score
GDS: Global deficit score
MO: Morbid obesity
WHR: Waist-to-hip ratio
